# FGF2 cooperates with IL-17 to promote autoimmune inflammation

**DOI:** 10.1038/s41598-017-07597-8

**Published:** 2017-08-01

**Authors:** Xinrui Shao, Siyuan Chen, Daping Yang, Mengtao Cao, Yikun Yao, Zhengxi Wu, Ningli Li, Nan Shen, Xiaoxia Li, Xinyang Song, Youcun Qian

**Affiliations:** 1 The Key Laboratory of Stem Cell Biology, Institute of Health Sciences, Shanghai Jiao Tong University School of Medicine/Shanghai Institutes for Biological Sciences, Chinese Academy of Sciences, Shanghai, 200025 China; 2 Shanghai Institute of Rheumatology, Shanghai Renji Hospital, Shanghai Jiaotong University School of Medicine, Shanghai, 200001 China; 3 Shanghai Institute of Immunology, Institute of medical sciences, Shanghai Jiao Tong University School of Medicine, Shanghai, 200025 China; 4 Department of Immunology, Lerner Research Institute, Cleveland Clinic Foundation, Cleveland, OH 44195 USA; 5 Division of Immunology, Department of Microbiology and Immunobiology, Harvard Medical School, Boston, MA 02115 USA

## Abstract

IL-17 is a pro-inflammatory cytokine implicated a variety of autoimmune diseases. We have recently reported that FGF2 cooperates with IL-17 to protect intestinal epithelium during dextran sodium sulfate (DSS)-induced colitis. Here, we report a pathogenic role of the FGF2-IL-17 cooperation in the pathogenesis of autoimmune arthritis. Combined treatment with FGF2 and IL-17 synergistically induced ERK activation as well as the production of cytokines and chemokines in human synovial intimal resident fibroblast-like synoviocytes (FLS). Furthermore, ectopic expression of FGF2 in mouse joints potentiated IL-17-induced inflammatory cytokine and chemokine production in the tissue. In the collagen-induced arthritis (CIA) model, while ectopic expression of FGF2 *in vivo* exacerbated tissue inflammation and disease symptom in the wild-type controls, the effect was largely blunted in *Il17a* ^−/−^ mice. Taken together, our study suggests that FGF2 cooperates with IL-17 to promote the pathogenesis of autoimmune arthritis by cooperating with IL-17 to induce inflammatory response.

## Introduction

Rheumatoid arthritis (RA) is a common and disabling autoimmune disease with pathology mostly in diarthrodial joints of the feet and hands^1, 2^. While the etiology remains unclear, the pathogenesis of RA is associated with chronic inflammation in the synovial tissues, which leads to cartilage disruption and bone erosion^3, 4^.

T cells have long been considered to contribute to the pathogenesis of RA^5, 6^. Recent studies have further defined the Th17 cells as a major T helper cell subset in promoting chronic inflammation in RA^7, 8^. IL-17A (also named IL-17), a Th17 signature cytokine, is increased in synovial fluids of RA patients^9, 10^. Previous studies have demonstrated that impairment of Th17 function or ablation of IL-17 signaling significantly delays the onset and reduces the severity of disease in collagen-induced arthritis (CIA), a mouse model for RA^7, 11–13^. Importantly, anti-IL-17 and anti-IL-17RA neutralizing antibodies have also shown promising efficacy in treating RA in clinical trials^14–17^.

IL-17 can induce the production of pro-inflammatory cytokines and chemokines alone or in cooperation with other cytokines such as TNFα^13^. IL-17 stimulation activates TRAF6-dependent NF-κB and MAPKs activation^18^ for gene transcription; it also engages a TRAF6-independent, TRAF2/5-dependent signaling for mRNA stabilization^19^. Act1 is an essential adaptor molecule bridging the IL-17 receptors to the downstream signaling^20, 21^. Similar to the *Il17a*-deficient mice, CIA pathogenesis is dramatically reduced in *Act1*-deficient mice^22^. IL-17 signaling has been shown to be tightly regulated to prevent excessive inflammation^23–26^.

In addition to IL-17, the growth factor FGF2 is also increased in RA patients and its level strongly correlates with Larsen’s grade of bone erosion^27, 28^. FGF2 blocking antibody significantly reduces adjuvant-induced arthritis (AIA) pathogenesis in rat^29^, suggesting FGF2 may be important for joint inflammation. However, it is not clear how FGF2 functions during the autoimmune pathogenesis. FGF2 signals through its receptor FGFR1. FRS2 (the FGF receptor substrate 2) binds to FGFR1 and recruits the downstream constitutive associated Grb2-Sos1 complex to activate the ERK pathway^30–32^. FRS2 also binds to SHP2 to form a complex with Grb2 to mediate FGF2-induced ERK signaling^32^.

We previously reported that IL-17 cooperates with FGF2 to induce ERK activation and target gene expression, a process critical for the repair the damaged intestinal epithelium to protect against dysregulated microbiota-driven colitis^33^. Here we found that FGF2 also synergized with IL-17 to induce ERK activation to promote autoimmune inflammation during the pathogenesis of arthritis.

## Results

### Both FGF2 and IL-17 are up-regulated in RA and CIA samples

**Figure 1:**
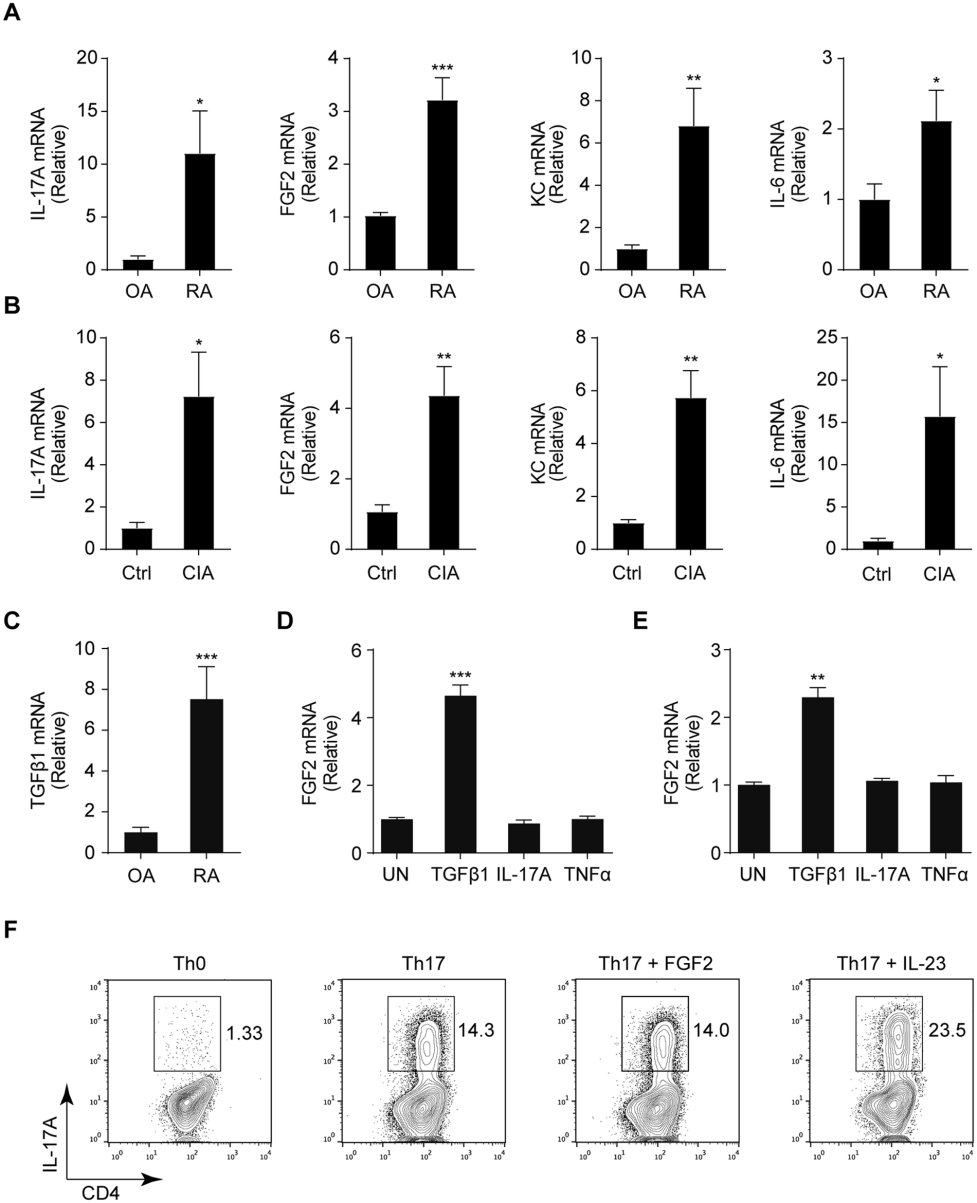
FGF2 and IL-17 are simultaneously elevated in the synovia of RA patients and the joints of CIA model. (**A**) Quantitative mRNA expression of IL-17, FGF2, IL-6 and KC in the synovia from 13 individuals with rheumatoid arthritis (RA) and 12 individuals with osteoarthritis (OA). (**B**) Quantitative mRNA expression of IL-17, FGF2, IL-6 and KC in in joints of wild type C57/BL6 left untreated or treated with the condition for CIA (n = 4). (**C**) Quantitative mRNA expression of TGFβ1 in the synovia from 13 individuals with RA and 12 individuals with OA as in (**A**). (**D** and **E**) Quantitative mRNA expression of FGF2 in human primary FLS cells (**D**) and MEFs (**E**) left untreated (UN) or stimulated for 6 hr with TGFβ1 (20 ng/ml), IL-17A (100 ng/ml) or TNFα (20 ng/ml). Cells were serum-starved for 16 hr before treatment. (**F**) IL-17A levels in *in vitro*-differentiated Th17 cells by TGFβ1 (2 ng/ml) and IL-6 (40 ng/ml), with or without FGF2 (20 ng/ml) or IL-23 (20 ng/ml). Data are representative of three independent experiments in (**B**, **D**–**F**). Data represent means and s.e.m. in (**A**–**E**) *P < 0.05, **P < 0.01, ***P < 0.001 by Student’s t test.

FGF2 and IL-17 have been separately shown to be elevated in the synovial fluids from RA patients^9, 10, 27, 28^. To examine the possible cooperation between IL-17 and FGF2 in the pathogenesis of RA, we measured IL-17 and FGF2 levels in the synovium sample from the same RA patients. The result revealed a concurrent up-regulation of FGF2 and IL-17 in the samples from RA patients (Fig. 1A). Both FGF2 and IL-17 are critical for the pathogenesis of inflammatory tissue destruction in rodent models of RA^11, 29^. Collagen-induced arthritis (CIA) is one of the most widely used mouse models for modeling RA pathology^34–37^. We found FGF2 and IL-17 were concurrently up-regulated in the joint tissues from mice on the CIA model (Fig. 1B). These data suggest that FGF2 and IL-17 may cooperate to mediate the pathogenesis of RA.

We previously reported that TGFβ induced FGF2 expression in *in vitro* differentiated CD4^+^ T cells such as Th17 and Treg cells^33^. Here we found that TGFβ expression was also up-regulated in the RA samples (Fig. 1C), suggesting that TGFβ also could induce FGF2 expression in infiltrated CD4^+^ T cells in the autoimmune disease. In addition to T cells, TGFβ induced weak FGF2 expression in resident colonic epithelial cells^33^. To check potential induction of FGF2 expression in RA resident cells, we purified synovial intimal resident fibroblast-like synoviocytes (FLS) from RA patients, and found that TGFβ but not IL-17 or TNFα induced FGF2 expression in these cells (Fig. 1D and Supplementary Fig. S1) while all the cytokines induced IL-6 expression (Supplementary Fig. S2). Similarly, TGFβ but not IL-17 or TNFα induced FGF2 expression in mouse embryonic fibroblasts (MEFs) (Fig. 1E and Supplementary Fig. S2). These data suggest that TGFβ could induce FGF2 expression in multiple cell types in RA pathogenesis. Furthermore, we found that FGF2 did not promote Th17 development for IL-17 production *in vitro* (Fig. 1F). Thus, our data suggest that FGF2 and IL-17 do not directly induce each other’s expression.

### FGF2 synergizes with IL-17 to induce cytokines and chemokines

**Figure 2:**
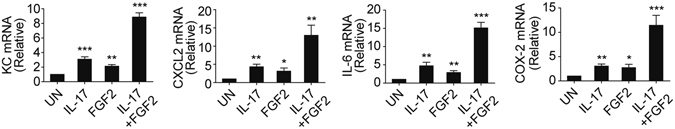
FGF2 synergizes with IL-17 to induce pro-inflammatory gene production. Quantitative mRNA expression of KC, CXCL2, IL-6 and COX-2 in human primary FLS cells left untreated (UN) or stimulated for 6 hr with IL-17 (50 ng/ml), FGF2 (5 ng/ml) alone or with FGF2 plus IL-17. Data are representative of three independent experiments (means and s.e.m.). *P < 0.05, **P < 0.01, ***P < 0.001 by Student’s t test.

**Figure 3:**
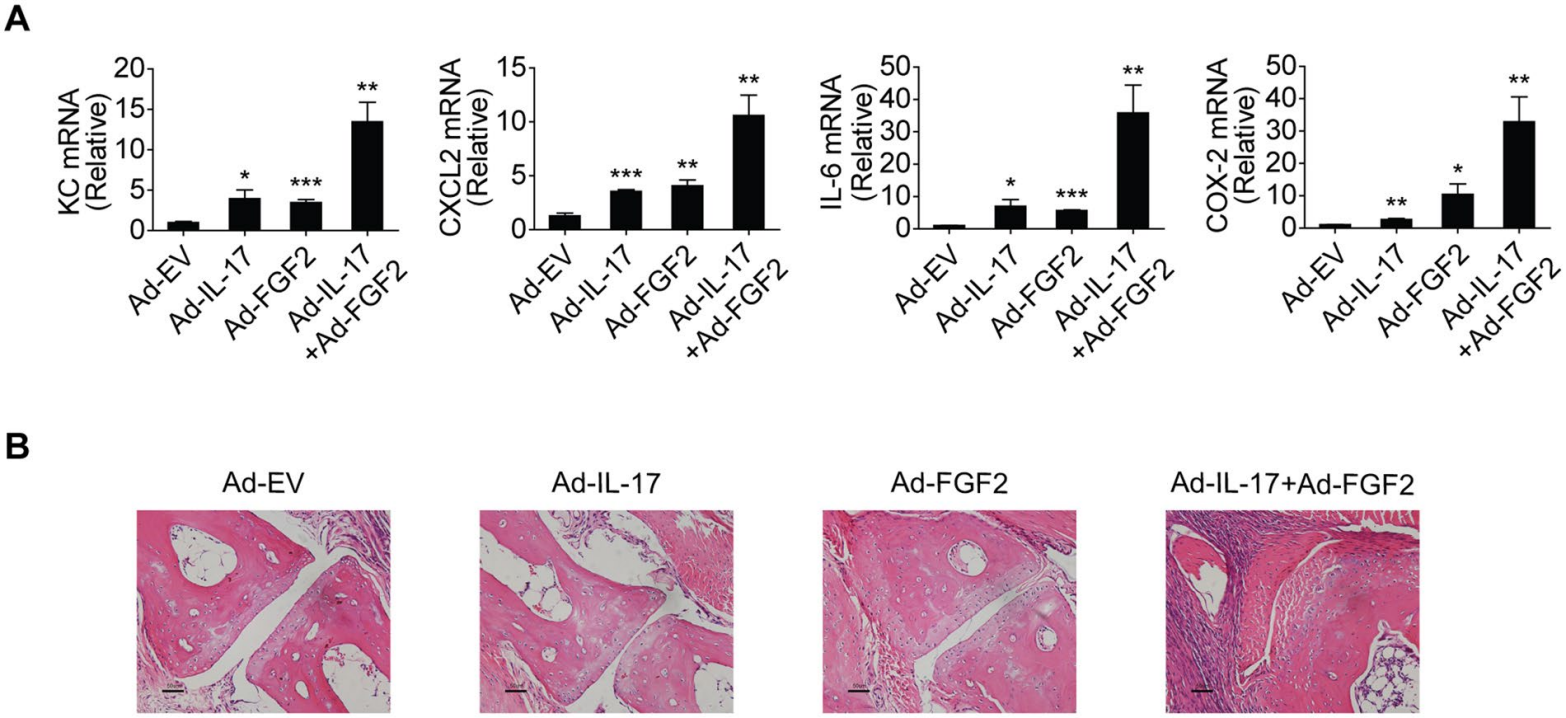
FGF2 synergizes with IL-17 to promote inflammatory pathogenesis. (**A**) Quantitative mRNA expression of KC, CXCL2, IL-6 and COX-2 in the joints of C57/BL6 mice treated with empty virus (Ad-EV), adenovirus expressing IL-17 (Ad–IL-17), adenovirus expressing FGF2 (Ad–FGF2) or adenovirus expressing IL-17 plus adenovirus expressing FGF2 for multiple times as indicated (n = 4). (**B**) H&E histology of the representative ankle sections from C57/BL6 mice treated as in (**B**). Data are representative of three (**A** and **B**) independent experiments (mean and s.e.m. in **A**). *P < 0.05, **P < 0.01, ***P < 0.001 by Student’s t test.

Synovial intimal resident fibroblast-like synoviocytes (FLS) are major sources of pro-inflammatory cytokines/chemokines and critically contribute to cartilage destruction^38^. We tested the FLS responses to FGF2 and IL-17 stimulation. While FGF2 or IL-17 alone exhibited limited effect on the expression of the tested pro-inflammatory cytokines and chemokines in the FLS, combined stimulation with FGF2 and IL-17 synergistically induced production of KC, CXCL2, IL-6, and COX-2 (Fig. 2). To determine whether FGF2 cooperates with IL-17 *in vivo*, we injected adenoviruses expressing FGF2 and/or IL-17 into the joints of healthy mice. Consistent with *in vitro* data, FGF2 synergized with IL-17 to induce proinflammatory genes production in mouse joint tissues (Fig. 3A). Histology analysis showed that simultaneous expression of both FGF2 and IL-17 led to severer tissue swelling and immune cell infiltration than that induced by either cytokine alone (Fig. 3B). These results suggest that FGF2 and IL-17 may synergistically promote joint inflammation.

### FGF2 cooperates with IL-17 to promote autoinflammatory pathogenesis

**Figure 4:**
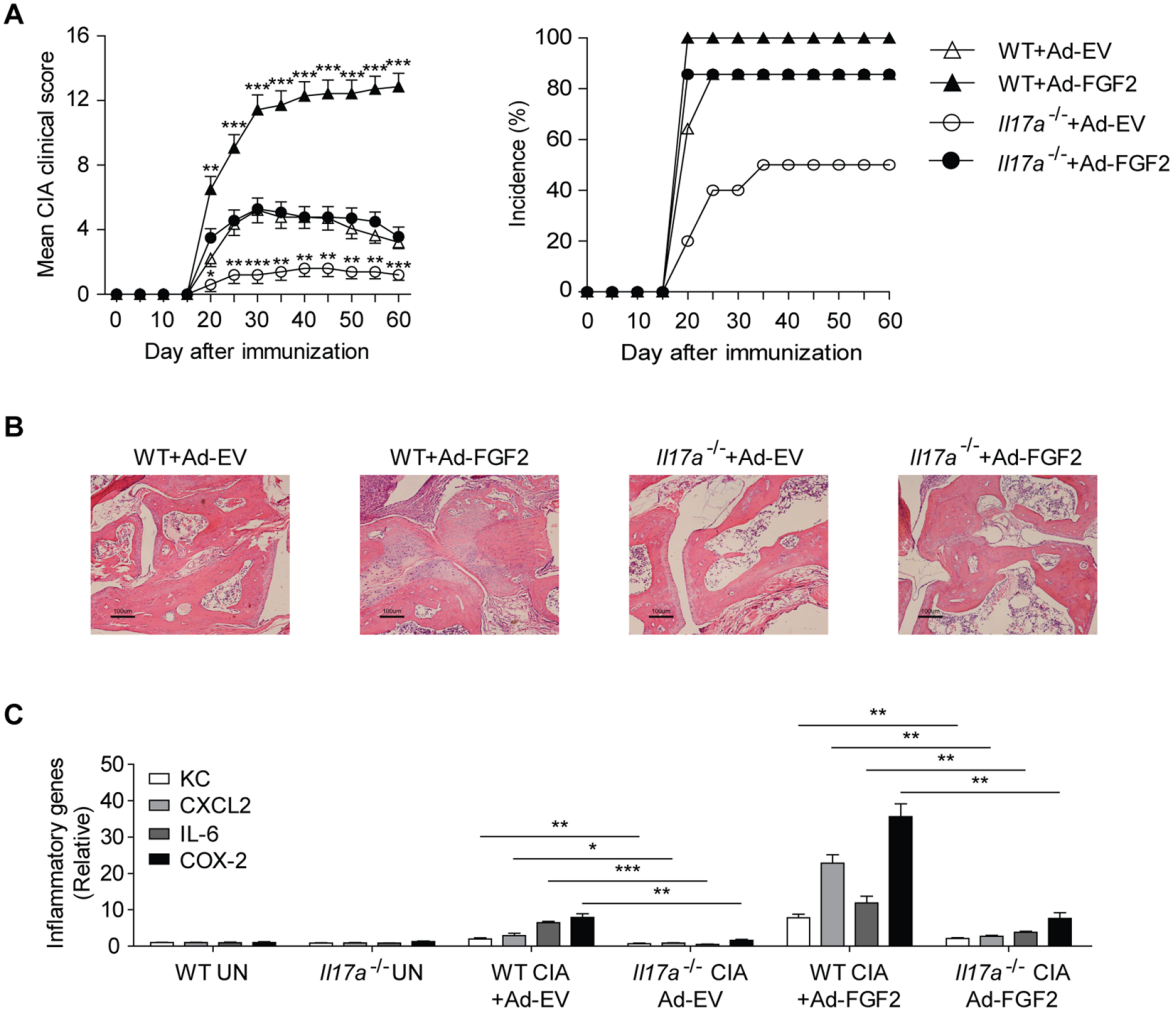
FGF2 cooperates with IL-17 to promote CIA pathogenesis. (**A**) Mean clinical scores and disease incidence of CIA in C57/BL6 wild type mice or *Il17a* ^−/−^ mice treated with empty virus (Ad-EV) or adenovirus expressing FGF2 (Ad–FGF2) once a week from day 15 after CIA induction (n = 10–14 per group). (**B**) H&E histology of the representative ankle sections from C57/BL6 wild type or *Il17a* ^−/−^ mice treated as in (**A**). (**C**) Quantitative mRNA expression of KC, CXCL2, IL-6 and COX-2 in the joints of C57/BL6 wild type or *Il17a* ^−/−^ mice treated as in (**A**) (n = 3–4 per group). Data are representative of two (**A**–**C**) independent experiments (mean and s.e.m. in **A**,** C**). *P < 0.05, **P < 0.01, ***P < 0.001 by Student’s t test.

To determine the role of the FGF2-IL-17 cooperation during the pathogenesis CIA, we injected FGF2-expressing adenovirus into the joints of *Il17a*-deficient and wild-type control mice on the CIA model. Consistent with previous report^11^, IL-17 deficiency reduced CIA clinical score and attenuated the joint tissue damage (Fig. 4A, B). Importantly, we found that ectopic expression of FGF2 severely aggravated the clinical symptoms and exacerbated the joint inflammation in the wild-type mice (Fig. 4A, B). The effect from FGF2 expression was greatly reduced in *Il17a*-deficient mice (Fig. 4A, B). In addition, IL-17 deficiency also attenuated FGF2-mediated increase of inflammatory cytokine and chemokine production (Fig. 4C). Together, these data suggest FGF2 cooperates with IL-17 to promote CIA pathogenesis.

### Act1 negatively regulates FGF2-induced ERK signaling in human primary FLS cells

**Figure 5:**
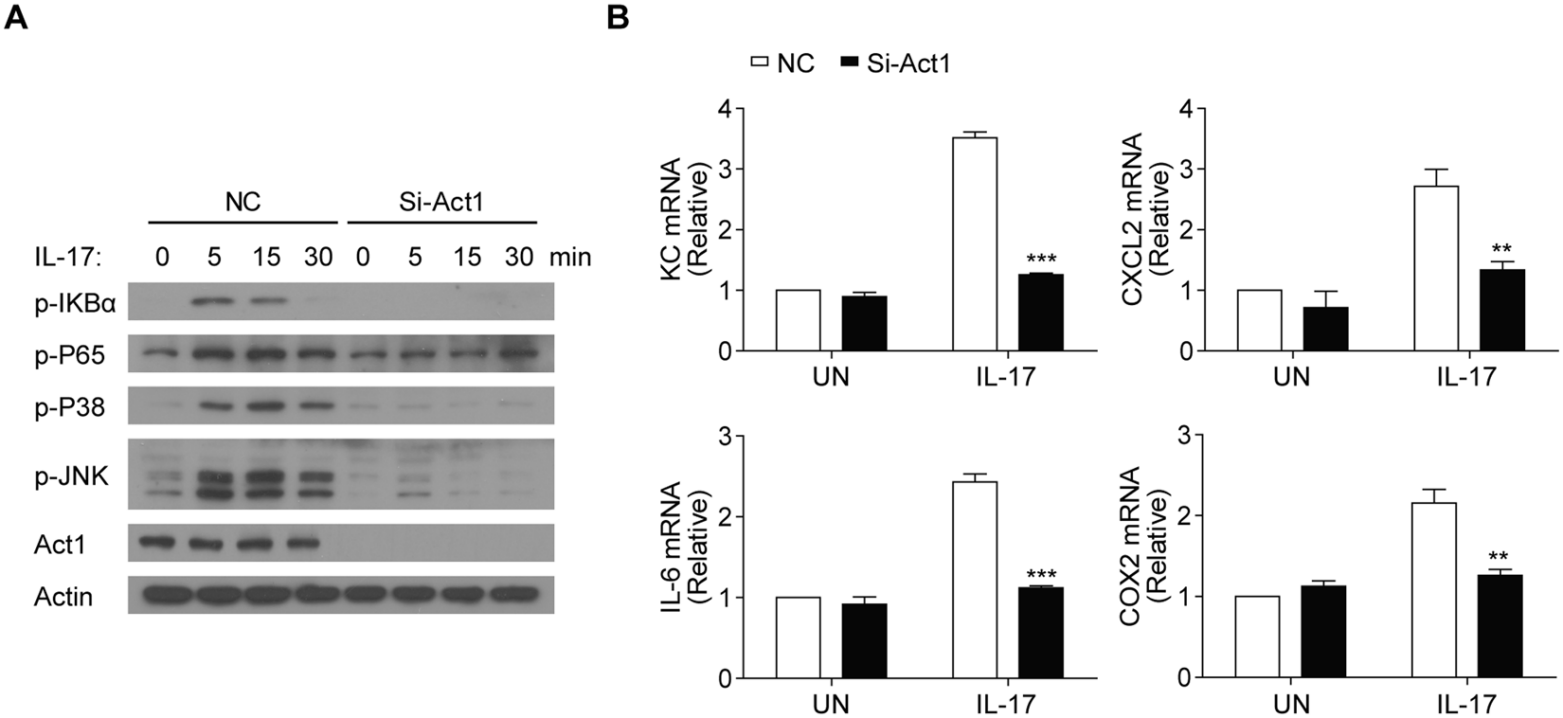
Act1 is required for IL-17 signaling in FLS cells. (**A**) Western blot analysis of phosphorylated (p-) or total proteins with the indicated antibodies in lysates of human primary FLS cells infected with control virus (NC) or the virus for Act1-specific siRNA (Si-Act1) and then left untreated (0) or treated for the indicated time points with IL-17 (50 ng/ml). (**B**) Quantitative mRNA expression of KC, CXCL2, IL-6 and COX-2 in the FLS cells infected as in (**A**) and then left untreated (0) or treated for 1 hr with IL-17 (50 ng/ml). Data are representative of three independent experiments (mean and s.e.m. in **B**). **P < 0.01, ***P < 0.001 by Student’s t test.

**Figure 6:**
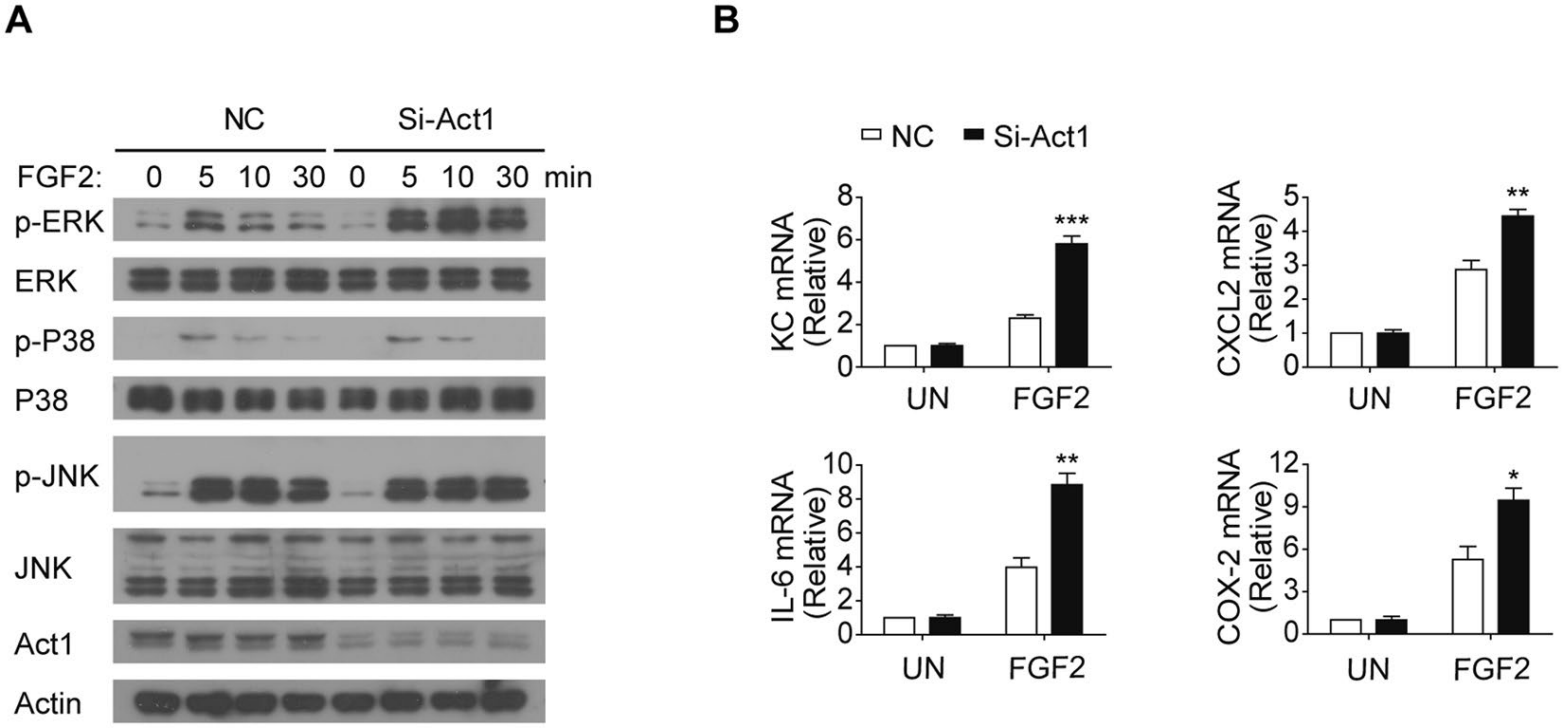
Act1 suppresses FGF2-mediated ERK activation. (**A**) Western blot analysis with antibodies against the indicated phosphorylated (p-) or total proteins in lysates of human primary FLS cells infected with control lentivirus or the lentivirus for Act1-specific siRNA (Si-Act1), and then left untreated (0) or treated for 5 to 30 min with FGF2 (50 ng/ml). (**B**) Quantitative mRNA expression of KC, CXCL2, IL-6 and COX-2 in human primary FLS cells infected as in (**A**) and then left untreated (0) or treated for 1 hr with FGF2 (50 ng/ml). Data are representative of three independent experiments (mean and s.e.m. in **B**). *P < 0.05, **P < 0.01, ***P < 0.001 by Student’s t test.

Act1 is the adaptor molecule for IL-17 signaling. siRNA-mediated knockdown of Act1 expression in the human primary FLS cells abolished IL-17-induced phosphorylation of IκBα, diminished MAPKs activation (Fig. 5A) and blocked IL-17-mediated expression of pro-inflammatory genes (Fig. 5B). In contrast to the positive role of Act1 in IL-17 signaling, we have previously shown that Act1 negatively regulates FGF2-induced ERK signaling in epithelial cells^33^. Consistently, we found that FGF2-induced ERK activation was enhanced in FLS cells transfected with Act1-targeting siRNA compared to the control siRNA transfected cells (Fig. 6A). Moreover, knocking down of Act1 increased FGF2-induced expression of KC and IL-6 (Fig. 6B). Of note, FGF2-induced activation of JNK and P38 was not affected by the Act1-knockdown (Fig. 6A), suggesting a specific regulation of FGF2-induced ERK signaling by Act1.

**Figure 7:**
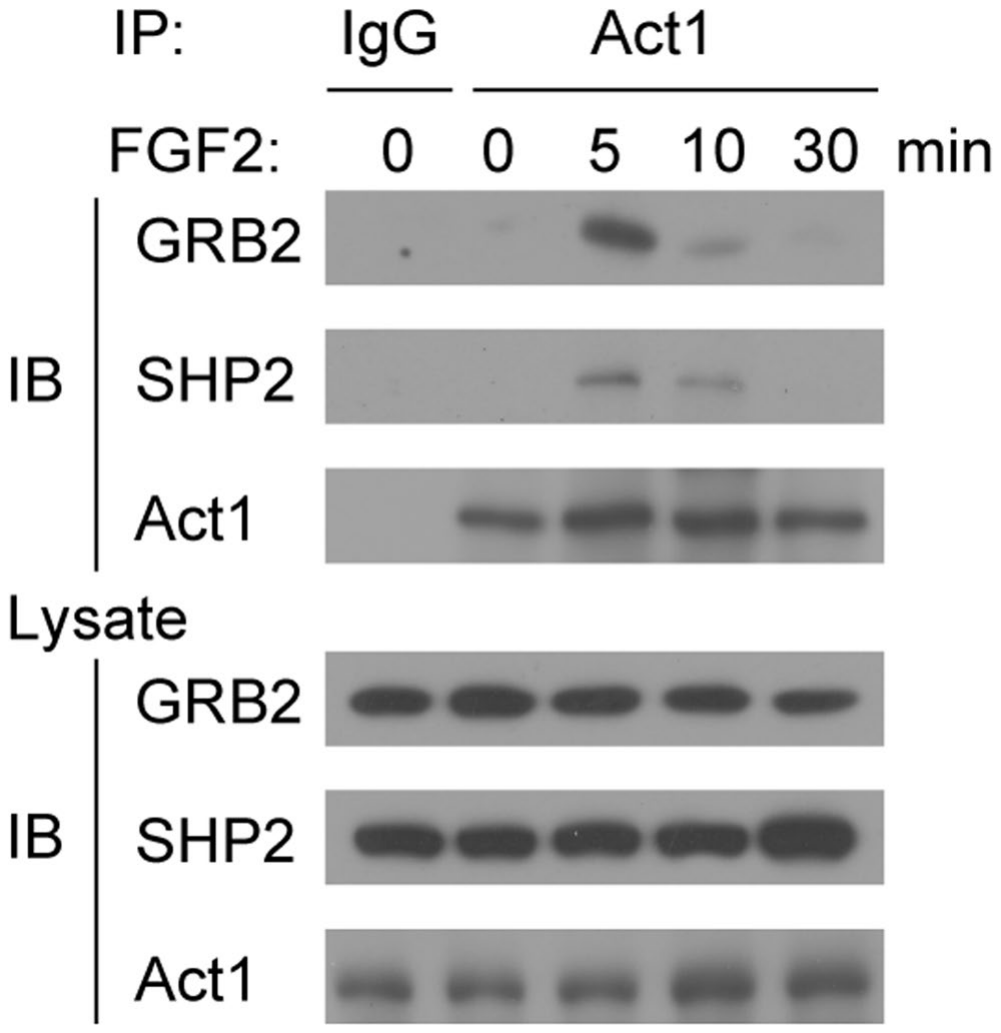
Act1 is recruited to Grb2 complex in FLS cells after FGF2 stimulation. Co-immunoprecipitation of cell lysates from human primary FLS cells. Cells were left untreated (0) or treated for the indicated time points with FGF2 (50 ng/ml). Whole cell lysates were immunoprecipitated with anti-Act1 and immunoblotted with the indicated antibodies. Data are representative of two independent experiments.

We have previously shown that Act1 suppresses FGF2-induced ERK activation by competing with SOS1 for Grb2 binding^33^. Similarly, we observed that Act1 associated with Grb2 and its signaling partner SHP2 upon FGF2 stimulation in the human primary FLS cells (Fig. 7). These results suggest that Act1 is recruited to FGF2-induced signaling complex to suppress its downstream ERK signaling in human FLS cells.

### FGF2 cooperates with IL-17 to mediate ERK activation for synergistic induction of pro-inflammatory genes

**Figure 8:**
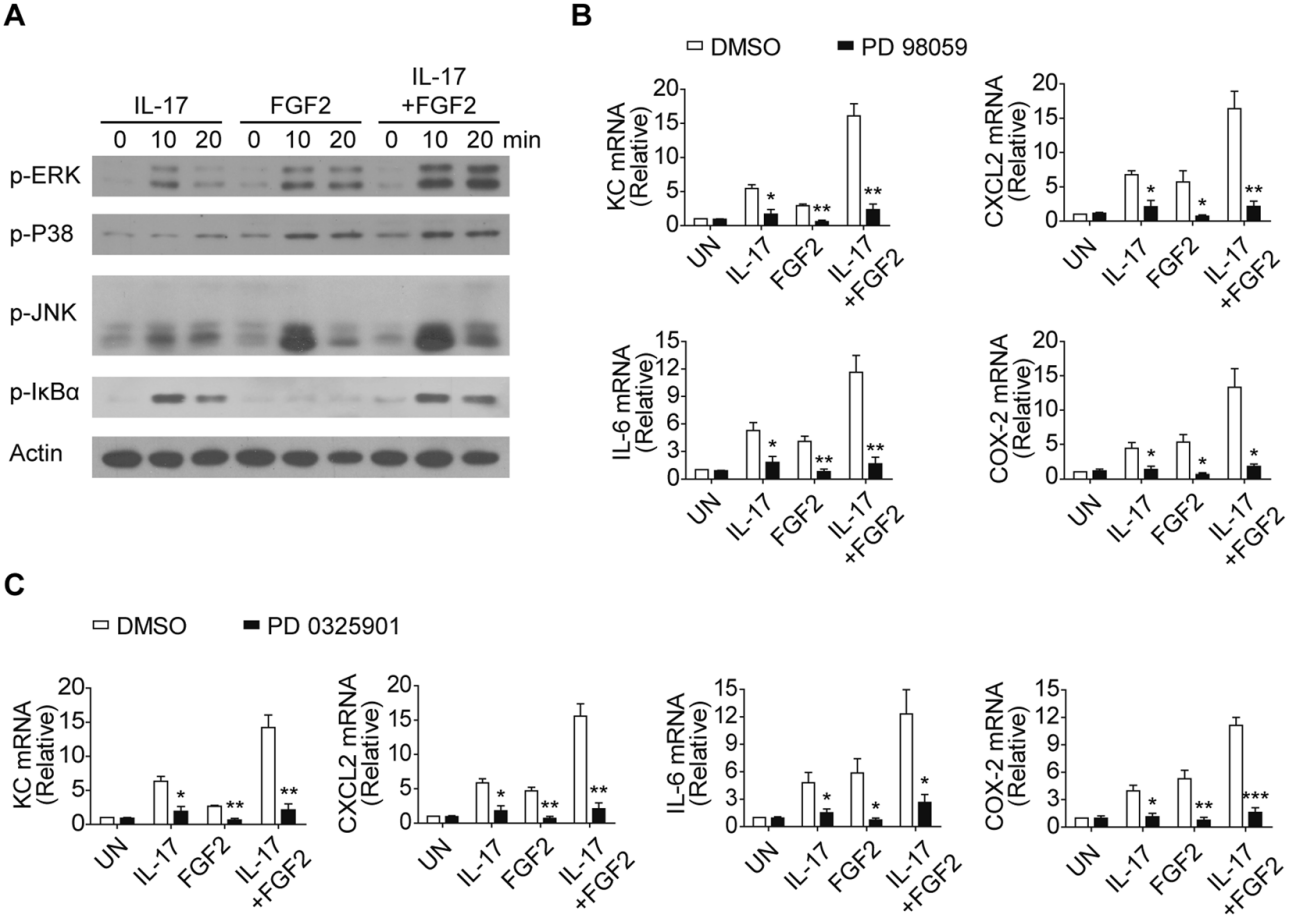
FGF2 synergizes with IL-17 to activate ERK signaling. (**A**) Western blot analysis of phosphorylated- (p-) Erk, p-P38, p-JNK, p-IκBα and Actin in lysates of human primary FLS cells left untreated (0) or treated for 10 and 20 min with IL-17 (50 ng/ml), FGF2 (5 ng/ml) alone or FGF2 plus IL-17. (**B** and **C**) Quantitative mRNA expression of KC, CXCL2, IL-6 and COX-2 in human primary FLS cells left untreated (UN) or stimulated for 6 hr with IL-17 (50 ng/ml), FGF2 (5 ng/ml) alone or FGF2 plus IL-17 in the present of DMSO, or DMSO dissolved Erk inhibitors PD 98059 (20 μM) (**B**) or PD 0325091 (20 μM) (**C**). Data are representative of three independent experiments (mean and s.e.m. in **B** and **C**). *P < 0.05, **P < 0.01, ***P < 0.001 by Student’s t test.

To determine the signaling pathway that mediates the IL-17-FGF2 cooperation in FLS cells, we examined the activation of NF-κB and MAPKs (JNK, P38 and ERK) in response to the stimulation with FGF2, IL-17 or FGF2 plus IL-17. Interestingly, only ERK signaling was synergistically activated by IL-17 and FGF2 in the FLS cells (Fig. 8A), reminiscent of our previous observation in epithelial cells^33^. Importantly, pharmacological inhibition of ERK activation using the MEK inhibitor PD 98059 drastically reduced the pro-inflammatory cytokine and chemokine induction induced by IL-17 and FGF2 treatment (Fig. 8B). The result was further confirmed by a second inhibitor, PD 0325901, which also suppresses the phosphorylation of ERK1/2 (Fig. 8C). Taken together, these results suggest that FGF2 synergizes with IL-17 to induce pro-inflammatory cytokine and chemokine expression via ERK1/2 activation.

## Discussion

FGF2 is up-regulated in RA patients and critical for the pathogenesis of AIA^27–29^. However, it remains elusive how the growth factor contributes to the inflammatory autoimmune pathogenesis. Here we demonstrate that FGF2 cooperates with a proinflammatory cytokine IL-17 to synergistically promote autoimmune inflammation. We found that FGF2 and IL-17 were simultaneously up-regulated in the joint tissues of RA as well as CIA. TGFβ was similarly up-regulated in RA samples. We then found that TGFβ induced FGF2 expression in multiple RA relevant cell types including T cells, fibroblasts, and fibroblast-like synoviocytes. We also provided evidence that IL-17 and FGF2 did not induce each other’s expression directly *in vitro*. However, it is possible that FGF2 may indirectly promote IL-17 expression through inducing IL-6 expression *in vivo* since IL-6 signaling is critical for Th17 development. We further demonstrated that IL-17 and FGF2 synergistically induced pro-inflammatory genes production in FLS cells and in mice joints *in vivo*. Importantly, FGF2-mediated effects on the inflammatory pathogenesis of CIA were greatly diminished in *Il17a*-deficient mice, supporting the cooperation of FGF2 with IL-17 during CIA pathogenesis.

Both the adaptor protein Grb2 and the phosphatase SHP2 are critical for FGF2-induced Ras-ERK signaling^39^. Grb2 constitutively associates with Sos1, a guanine nucleotide exchange factor (GEF) for Ras. We have previously demonstrated that Act1 directly associates with Grb2 and interfere with FGF2-induced Grb2-Sos1 complex for ERK activation. Act1 is preferentially recruited to IL-17 receptor complex, which releases Act1-mediated suppression on FGF2-induced ERK signaling during co-stimulation of FGF2 and IL-17^33^. Similarly, here we found that FGF2 induced the association of Act1 with Grb2 and SHP2 in human primary FLS cells and that Act1 suppressed FGF2-induced ERK signaling and production of downstream pro-inflammatory genes in the FLS cells. The data suggest a similar molecular mechanism of Act1-mediated autoimmune inflammation driven by FGF2 and IL-17.

The FLS cells are reported to be the major cell type responsible for autoimmune inflammation^38^. We utilized the primary FLS cells to investigate the cooperation of FGF2 and IL-17 in promoting inflammation. Because many cell types are responsive to FGF2 and IL-17, our study does not exclude the contribution of IL-17-FGF2 cooperativity in other cell types during the pathogenesis of CIA and RA. Given the wide implication of IL-17 in autoimmune disease, the IL-17-FGF2 cooperation may also contribute to inflammatory pathology of other inflammation-driven autoimmune diseases such as psoriasis.

## Methods

### Human samples

Tissue specimens from arthritic joints of patients with osteoarthritis or rheumatoid arthritis were obtained as described^40^. Osteoarthritis and rheumatoid arthritis were diagnosed in accordance with the criteria of the American College of Rheumatology. All individuals provided informed consent. The study was approved by the Research Ethics Board of Shanghai Guanghua Hospital and the associated methods were performed in accordance with the relevant guidelines and regulations.

### Mice

*Il17a* ^−/−^ mice on the C57BL/6 background were kindly provided by Dr. Yoichiro Iwakura (University of Tokyo). *Il17a* ^−/−^ and its representative littermate control mice were utilized for experiments. All above mice were maintained in specific pathogen-free conditions. All animal experiments were performed in compliance with the guide for the care and use of laboratory animals and were approved by the institutional biomedical research ethics committee of the Shanghai Institutes for Biological Sciences (Chinese Academy of Sciences).

### Induction of CIA

8–10 weeks old *Il17a* ^−/−^ and its representative littermate control mice were injected intradermally with 100 μg emulsified CII (Sigma-Aldrich) plus CFA at several sites of the tail base on day 1 and day 21. Mice were observed every 5 d for scoring of clinical signs, and the observers were blind to evaluate the clinical scores.

### Histology

Joint tissues for histological analyses were dissected from CII-immunized mice and immediately fixed with 4% paraformaldehyde. Paraffin-embedded sections of joint were stained with H&E and then examined by light microscopy.

### Cell culture

Rheumatoid arthritis derived human fibroblast like synoviocytes (FLS) were kindly provided by Dr. Ningli Li (Shanghai Jiaotong University School of Medicine). The FLS cells, MEF cells, and 293A cells were grown in DMEM medium supplemented with 10% (vol/vol) FBS, penicillin (100 U/ml) and streptomycin (100 μg/ml). 293FT cells were grown in DMEM medium supplemented with 10% (vol/vol) FBS, 1% (vol/vol) NEAA, penicillin (100 U/ml) and streptomycin (100 μg/ml).

### *In vitro* generation of Th17 cells and flow cytometry

Naive CD4^+^ T cells were purified by magnetic sorting from spleens of C57BL/6 mice. Sorted cells were activated with pre-coated anti-CD3 (5 μg/ml) and soluble anti-CD28 (2 μg/ml) and were then induced to differentiate into Th0 and Th17 cells by adding various cytokines and antibodies as follows: Th0 condition, anti-IFNγ (10 μg/ml) and anti-IL-4 (10 μg/ml); Th17 condition, anti-IFNγ (10 μg/ml), anti-IL-4 (10 μg/ml), TGFβ1 (2 ng/ml) and IL-6 (40 ng/ml), with or without IL-23 (20 ng/ml). Cells were cultured with RPMI-1640 medium containing 10% (vol/vol) FBS, 2-mercaptoethanol (55 μM), penicillin (100 U/ml), and streptomycin (100 μg/ml). Fluorescence-labelled anti-mouse CD4-FITC (GK1.5) and anti-mouse IL-17A-PE (TC11-18H10) were obtained from BD Biosciences. All antibodies were used at 1:100 dilutions. Intracellular staining for IL-17A was performed as follows: differentiated CD4^+^ T cells were cultured in RPMI-1640 medium containing 10% FBS, 1% L-glutamine, penicillin (100 U/ml), and streptomycin (100 μg/ml) for 4 hours at 37 °C with phorbol 12-myristate 13-acetate (50 ng/ml; Sigma), ionomycin (500 ng/ml; Sigma), and brefeldin A (10 μg/ml; biolegend). After staining with CD4, cells were fixed and permeabilized by using Cytofix/Cytoperm solution (BD Biosciences) to perform the indicated intracellular cytokine staining. FACS Calibur (BD Biosciences) and FlowJo software were used for data acquiring and analysis.

### RNA isolation and real-time quantitative PCR

Total RNA was extracted from the indicated cells or mouse joint tissues with TRIzol reagent (Invitrogen) according to the manufacturer’s instructions. RNA samples were reverse-transcribed into cDNA by using a PrimeScript RT Reagent kit (TaKaRa). The cDNA samples were then amplified by quantitative PCR with a SYBR Premix ExTaq kit (TaKaRa) on an ABI PRISM 7900 HT cycler (Applied Biosystems). The expression of indicated genes was normalized to expression of housekeeping gene *Rpl13a*.

### Reagents

Recombinant FGF2 (100-18B) and recombinant TGFβ1 (100-21) were from PeproTech; recombinant IL-17A (7955-IL) and recombinant TNFα (210-TA**)** were from R&D Systems; Anti-Actin (A4700) were from Sigma. Antibody to Act1 (H300), GRB2 (C-23), phosphorylated Erk (sc-7383) was from Santa Cruz Biotechnology. Antibody to SHP2 (ab31110) was from abcam. Antibodies to phosphorylated IkBa (2859L), p65 (3033S), p38 (9211S) and Jnk (92516) were from Cell Signaling Technology. Antibodies to total Erk (9102), p38 (9212) and Jnk (9252) were also from Cell Signaling Technology. Erk inhibitors PD 98059 (P215) and PD 0325901 (PZ0162) were purchased from Sigma.

### Gene knockdown with lentivirus-delivered siRNA

The RNA interfere sequence for knockdown of human Act1 gene was 5′-GCTTCAGAACACTCATGTCTA-3′^25^; the scrambled sequence for control siRNA was 5′-GGATCCTTGACAATACCAA-3′^41^. The RNA interfere sequences were constructed into the lentivirus pLSLG vector. Lentivirus pLSLG vectors plus VSVG and delta 8.9 helper vectors were co-transfected into 293FT cells (Invitrogen) for lentiviral packaging. After transfection for 60 hours, virus particles were collected for infection of target cells in the presence of 10 mg/ml polybrene (Sigma). After 4 days of infection, cells were used for indicated treatments.

### Adenovirus-mediated gene expression in mice

Mouse IL-17 or FGF2 was constructed into the pAdTrack-CMV vector and then recombined with the pAdEasy-1 vector. Recombinant Ad-IL-17A, Ad-FGF2 or empty vector (Ad-EV) was transfected into QBI-HEK 293A cells (Qbiogene). Viruses were packaged and amplified as described^41^. After titration, 2 × 10^8^ adenovirus particles were injected into the joints of the indicated mice once a week for different experimental settings.

### Co-immunoprecipitation

The FLS cells were treated with or without FGF2, IL-17 or FGF2 plus IL-17 for the indicated time points. Cell extracts were incubated with 20 μl protein A beads (GE Healthcare) plus 0.5 μg of the antibodies against Act1. After overnight incubation at 4 °C, beads were washed with lysis buffer for four times and then analyzed by immunoblotting of indicated antibodies.

### Immunoblot analysis

Cells were directly lysed by Triton buffer (0.5% Triton X-100 and 20 mM HEPES, pH 7.6) on ice and the lysates were separated by 10% SDS-PAGE. Separated proteins were transferred onto polyvinylidene fluoride filters by using Mini Trans-Blot (Bio-Rad). The filters were then blocked with Tris-buffered saline with 5% non-fat milk plus 0.1% Tween 20 for 1 hour at room temperature. After blocking, the filters were incubated with primary antibody overnight at 4 °C and subsequently washed for three times with Tris-buffered saline containing 0.1% Tween 20. The filters were then incubated with HRP-conjugated secondary antibodies for 1 hour at room temperature. After washing for three times with Tris-buffered saline containing 0.1% Tween 20, indicated signals were detected by using chemiluminescent HRP substrate (Millipore).

### Statistics

Data are presented as mean ± s.e.m. A two-tailed Student’s t test was used for analysis of differences between the groups. P values of <0.05 were considered statistically significant.

## Electronic supplementary material

Supplementary figures 1 and 2
