# Supplementary material for: FGF2 cooperates with IL-17 to promote autoimmune inflammation
Source: Sci Rep. 2017 Aug 1;7:7024. doi: 10.1038/s41598-017-07597-8 (PMC5539112; doi:10.1038/s41598-017-07597-8)
Supplement: Supplementary file 1 — Supplementary figures 1 and 2 [file 41598_2017_7597_MOESM1_ESM.pdf]

Supplementary Information

Supplementary Figure S1 and S2

FGF2 cooperates with IL-17 to promote autoimmune inflammation

Xinrui Shao, Siyuan Chen, Daping Yang, Mengtao Cao, Yikun Yao, Zhengxi Wu, Ningli Li, Nan Shen, Xiaoxia Li, Xinyang Song, and Youcun Qian

Supplementary Figure S1

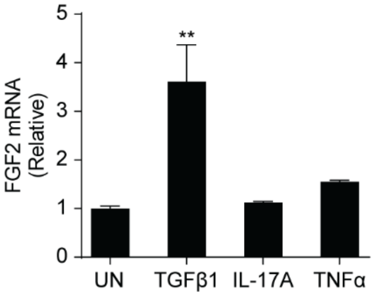

Supplementary Figure S1. FGF2 is induced by TGFβ1 in human primary FLS cells.

Quantitative mRNA expression of FGF2 in human primary FLS cells left untreated (UN) or stimulated for 24 hr with TGFβ1 (20 ng/ml), IL-17A (100 ng/ml) or TNFα (20 ng/ml). Cells were serum-starved for 16 hr before treatment. Data are representative of three independent experiments (means and s.e.m.). ** $P < 0.01$ by Student's t test.

Supplementary Figure S2

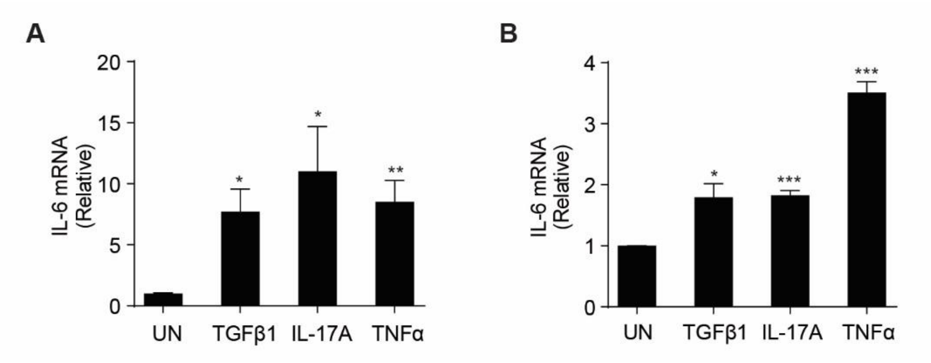

Supplementary Figure S2. IL-6 is induced by TGFβ1, IL-17 and TNFα in human primary FLS cells and MEFs.

(A and B) Quantitative mRNA expression of FGF2 in human primary FLS cells (A) and MEFs (B) left untreated (UN) or stimulated for 6 hr with TGFβ1 (20 ng/ml), IL-17A (100 ng/ml) or TNFα (20 ng/ml). Cells were serum-starved for 16 hr before treatment. Data are representative of three independent experiments (means and s.e.m.). *$P < 0.05$, ** $P < 0.01$, *** $P < 0.001$ by Student's t test.
